# Regional homogeneity of adolescents with high‐functioning autism spectrum disorder and its association with symptom severity

**DOI:** 10.1002/brb3.2693

**Published:** 2022-07-11

**Authors:** Xiaoxin Zhao, Shuyi Zhu, Yang Cao, Peipei Cheng, Yuxiong Lin, Zhixin Sun, Yan Li, Wenqing Jiang, Yasong Du

**Affiliations:** ^1^ Department of Child and Adolescent Psychiatry Shanghai Mental Health Center, Shanghai Jiao Tong University School of Medicine Shanghai China; ^2^ Department of Psychiatry Suzhou Guangji Hospital Suzhou China

**Keywords:** Autism Behavior Checklist, autism spectrum disorder, regional homogeneity, resting‐state functional magnetic resonance imaging, symptom severity

## Abstract

**Background and purpose:**

Previous studies have revealed abnormal regional homogeneity (ReHo) in individuals with autism spectrum disorder (ASD); however, there is little consistency across the findings within these studies, partly due to small sample size and great heterogeneity among participants between studies. Additionally, few studies have explored the association between ReHo aberrance and clinical symptoms in individuals with ASD.

**Methods:**

Forty‐eight adolescents with high‐functioning ASD and 63 group‐matched typically developing (TD) controls received functional magnetic resonance imaging at rest. Group‐level analysis was performed to detect differences in ReHo between ASD and TD. Evaluation of symptom severity in individuals with ASD was based on the Autism Behavior Checklist (ABC). Voxel‐wise correlation analysis was undergone to examine the correlations between the symptom severity and ReHo map in individuals with ASD within brain areas with ReHo abnormalities.

**Results:**

Compared with the TD controls, individuals with ASD exhibited increased ReHo in the bilateral anterior cingulate cortex, left caudate, right posterior cerebellum (cerebellar tonsil), and bilateral brainstem and decreased ReHo in the left precentral gyrus, left inferior parietal lobule, bilateral postcentral gyrus, and right anterior cerebellum (culmen). The correlation analysis indicated that the ReHo value in the brainstem was negatively associated with the ABC total scores and the scores of Relating factor, respectively.

**Conclusions:**

Our findings indicated that widespread ReHo abnormalities occurred in ASD, shedding light on the underlying neurobiology of pathogenesis and symptomatology of ASD.

## 1 INTRODUCTION

Autism spectrum disorder (ASD) is a severe and disabling neurodevelopmental disorder characterized by impairments in social interaction and communication, repetitive behaviors, and restricted interests (American Psychiatric Association, 2013). The estimated prevalence of ASD stands at approximately one in 54 children and is on the rise (Maenner et al., 2020). Besides, low self‐care ability and increased unemployment or underemployment are commonly present among patients with ASD when they reach adulthood (Strickland, 1997). With the high morbidity and disability, ASD poses a huge social and economic burden on families and caregivers of individual with ASD and even the whole of society. Unfortunately, the etiology and pathogenesis of ASD remain poorly understood and controversial.

However, it is noteworthy that neurological abnormalities have been considered as one of the most promising mechanism for the pathophysiology of ASD. A prior morphological study using structural magnetic resonance imaging (MRI) with numerous subjects ranging from toddlers to old adults reported ASD patients exhibited decreased gray matter volume (GMV) in the amygdala (van Rooij et al., 2018). Functionally, based on task‐based functional MRI, several studies demonstrated ASD‐related brain function deficits as characterized by atypical activation in social brain regions when processing social stimuli (Baron‐Cohen et al., 1999; Ciaramidaro et al., 2018; Muller & Fishman, 2018). For the past few years, resting‐state functional magnetic resonance imaging (rs‐fMRI) has received increasing attention for examining spontaneous brain activity in individuals with ASD.

It is widely acknowledged that the brain uses most of the energy at rest to support spontaneous activity, studies of which are expected to provide important information. Rs‐fMRI is considered as an effective method for investigating alterations of human brain function in the absence of tasks (Fox & Raichle, 2007). In recent years, there is increasing evidence that ASD is a disorder of disconnectivity. Previous studies indicated decreased resting‐state functional connectivity (FC) between the dorsomedial prefrontal cortex and posterior cingulate cortex within the default mode network in ASD patients (Padmanabhan et al., 2017; Pereira et al., 2018). Through graph theoretical analysis, Li et al. (2020) indicated that the nodal efficiency of the right lingual gyrus and the nodal degree of the right medial frontal gyrus in ASD patients were both larger than those of typically developing (TD) controls. Moreover, an independent component analysis study (Chen et al., 2020) demonstrated that compared with TD controls, children with ASD have increased intrinsic FC between visual and sensorimotor networks. Generally, such analytic methods as mentioned above for rs‐fMRI data focused on the long‐distance interregional temporal correlations of blood oxygen‐dependent level signals. However, these methods cannot fully characterize the local synchronization of voxels in certain brain regions. In recent years, regional homogeneity (ReHo) has been suggested as a promising method for measuring the local synchronization of neurobiological activity.

Assuming that a given voxel is temporally similar to its neighboring voxels, ReHo could be used to detect the metabolic activity synchronization of the voxels spurred by specific conditions without being disturbed by external stimuli, and for this reason, it gains an advantage over conventional MRI (Zang et al., 2004). Moreover, several research groups have proved ReHo is a local indicator with high retest reliability due to the high resistance to spatiotemporal noise and outliers (Zuo & Xing, 2014; Zuo et al., 2013), which is crucial on account of its direct involvement on its clinical applications. Specifically, increasing evidence is emerging to support the notion that ReHo metric has neurobiological relevance to anatomical, neurocognitive and behavioral factors, making it a potential biomarker for research into neuropsychiatric disorders. At present, the local metric has been successfully utilized in investigating local functional homogeneity in numerous neuropsychiatric diseases (An et al., 2013; Damiani et al., 2021; Jiang et al., 2020; Scalabrini et al., 2020; Zhao et al., 2019). Compared to studies with other neuropsychiatric disorders, however, relatively few attempts have been made to investigate individuals with ASD (Di Martino et al., 2014; Jiang et al., 2015; Maximo et al., 2013; Nair et al., 2017; Paakki et al., 2010; Shukla et al., 2010). These preliminary studies reveal the significance of ReHo analysis and the great potential for testing the short‐range disconnection in ASD, and how it can complement long‐distance connectivity analysis. However, these ReHo studies of ASD also showed inconsistent results, with both higher and lower ReHo in ASD group compared with TD group (Dajani & Uddin, 2016; Paakki et al., 2010; Shukla et al., 2010) with poor replication across these studies. Some studies showed higher ReHo values in the middle frontal gyrus (Jiang et al., 2015; Maximo et al., 2013), while other studies reported lower ReHo values in this area (Paakki et al., 2010; Shukla et al., 2010) in ASD. The inconsistent results of these studies may be due to the small sample size, differences in imaging methodology and demographic and clinical characteristics (age, gender, or intelligence quotient, etc.), and limited brain regions investigated. Small sample size limits the statistical power for investigation of comparison during study data analysis as it requires correction for multiple comparisons. Methodological differences may also have an effect on the measurement of GMV and lead to variable results, such as image acquisition and image analysis. As ASD is a heterogeneous developmental disorder (Chen et al., 2015), there are inherent differences between patients, with diverse clinical manifestations and different levels of intelligence, which is most likely associated with variations in neuroanatomical abnormalities. Thus, it has been proved that ReHo abnormalities are worth further investigation in ASD patients through the recruitment of relatively large‐size and homogeneous samples of patients, reducing the influence from potential confounding factors.

Many of previous studies investigating ReHo alterations in ASD have recruited young children (Ciarrusta et al., 2020; Lan et al., 2021), or mixed groups of participants at different age levels (Jao Keehn et al., 2019; Jiang et al., 2015). However, relatively few attempts have been made to investigate adolescents with ASD. The results have also been inconsistent. For example, Paakki et al. (2010) has reported numerous regions with both increased and decreased ReHo in adolescents with ASD. However, Dajani and Uddin (2016) failed to replicate the finding and found adolescents with ASD exhibited similar level of ReHo as age‐matched TD controls. Therefore, the central question of how the brain ReHo alterations manifest in adolescent ASD remains unaddressed and deserves further examination.

In addition to the ReHo alterations, the relationship between ReHo aberrance and clinical characteristics has also begun to attract increasing attention from researchers. A few pioneering studies have begun to investigate the potential association. Jiang et al. (2015) reported the ReHo in the right middle frontal gyrus correlated positively with the overall total scores on the autism diagnostic observation schedule (ADOS), and the ReHo in the right superior temporal sulcus correlated positively with scores of communication, stereotyped behaviors and restricted interests. However, Li et al. (2018) found that there was no correlation between ReHo and clinical symptoms in their research, and their sample is relatively small. It should be noted that the limited recruitment of patients with ASD and differences in sample characteristics are consequently presumed to be insufficient for the exploration of the association between ReHo metric and clinical symptoms. Given the insufficient examinations and inconsistent findings on the association between ReHo and symptom severity in ASD, we recruited a relatively large‐size and homogeneous sample to examine the association, making the findings of the study more robust. Moreover, the investigation of the relationship between abnormal behavioral performance and abnormal ReHo of ASD may be of great importance in enlightening the core neuropathology of this illness and may provide significant insight into the pathogenesis of the disease, its diagnosis, and the optimal path for therapeutic intervention.

In this study, we use ReHo measurement to examine the local brain function of adolescents with high‐functioning autism spectrum disorder (HFASD). With interest in the potential neuromechanism mediating clinical symptoms of ASD, we also tried to explore the correlation between the ReHo alterations and the clinical symptom, using the Autism Behavior Checklist (ABC) (Rellini et al., 2004). To address issues of sample variability and the inconsistency in neuroanatomical findings, we recruited a relatively larger sample and used strict inclusion criteria for individuals with ASD according to age, gender, intelligence quotient (IQ), and diagnosis, and TD controls matched for age, gender, and IQ. Based on the findings of previous studies, we hypothesized that (1) individuals with ASD would present ReHo alterations in specific brain regions, and (2) the alterations of ReHo in these brain regions would correlate with clinical symptoms of individuals with ASD.

## 2 METHODS

### 2.1 Subjects

Fifty‐eight adolescents with HFASD aged 10–18 years from Shanghai Mental Health Center, were recruited in the current study from February 2017 to May 2020. Sixty‐six TD controls were invited from the local community via advertisements. All of the patients were diagnosed using DSM‐5 (American Psychiatric Association, 2013), and fulfilled the criteria for ASD. None of the control participants presented current or past neurological or psychiatric disorders. Seven ASD participants and one control subject were excluded because of excessive movements. Additionally, three other ASD patients and two TD controls were excluded to create the matched groups matching on age, gender, and full‐scale intelligence quotient (FSIQ), resulting in a final sample of 48 ASD (39 males, 9 females) and 63 TD (43 males, 20 females) participants (Table 1). TD controls with first‐degree family history of psychotic episodes were also excluded. IQ was assessed using the Wechsler Intelligence Scale for Children‐IV (WISC‐IV; Grizzle, 2011) or the Wechsler Adult Intelligence Scale‐IV (WAIS‐IV; Benson et al., 2010), depending on the age of the teen. Every participant with ASD scored above the common cutoff for the distinction of “low” and “high” functioning ASD (FSIQ > 80) (Itahashi et al., 2015). All participants were Han Chinese, and classified as right‐handers according to the Annett Handedness Scale (Annett, 1967). The patients had a mean ± SD age of 13.0 ± 1.9 (range, 10–18) and a mean ± SD FSIQ of 106.9 ± 19.2 (range, 80–145). The controls had a mean ± SD age of 12.9 ± 1.8 years (range, 10–18) and a mean ± SD FSIQ of 106.9 ± 19.2 (range, 80–143).

**TABLE 1:** Demographic data and abnormal behavioral performance in individuals with ASD and TD controls

	ASD (*n* = 48)^a^		TD (*n* = 63)^a^		Statistics	
	Mean	SD	Mean	SD	*t*/*x* ^2^	*p* Value
Age, years	13.0	1.90	12.9	1.8	0.33	.74^b^
Gender (male/female)	39/9		43/20		2.384	.123^c^
Handedness (right/left)	48/0		63/0		–	–
FSIQ	106.9	19.2	111.0	14.3	–1.26	.211^b^
ABC						
Sensory	11.3	5.8	NA		–	–
Relating	19.9	7.3	NA		–	–
Body concept	13.0	8.7	NA		–	–
Language	13.2	8.1	NA		–	–
Social self‐help	14.1	5.6	NA		–	–
Total ABC score	71.42	28.3	NA		–	–

*Note*: There was no significant difference between ASD and TD control groups in age, gender, and FSIQ (all *p* > .05).

^a^ Seven ASD patients and one TD control were excluded due to excessive head motion. Three other ASD and two TD participants were excluded to restore group matching on age, gender, and FSIQ.

^b^ Two‐tailed *t*‐tests.

^c^ Two‐tailed chi‐square tests.

Abbreviations: ABC, Autism Behavior Checklist; ASD, autism spectrum disorder; FSIQ, full‐scale intelligence quotient; NA, not applicable; TD, typically developing.

General inclusion criteria for both groups were the following: (1) aged 10−18 years, (2) right‐handed, (3) full comprehension of the survey instructions and contents. All research procedures were conducted following the Declaration of Helsinki and were approved by the Medical Research Ethics Committee of Shanghai Mental Health Center. Written informed consent of the participants was obtained from his/her legally authorize guardians.

### 2.2 Psychopathological assessment

ABC is a checklist of nonadaptive behaviors (Marteleto & Pedromonico, 2005), which was designed as a clinical measure to more objectively identify autism in individuals 3–35 years of age. The ABC was completed by parents. The ABC consists of 57 items and differences in behaviors were noted in the five areas of development, including Sensory, Relating, Body concept, Language, and Social self‐help (Szatmari et al., 2003). ABC items were rated as “yes” (rated as 1, with symptom) or “no” (rated as 0, without symptom) for each question during the assessment. ABC was well‐established for the screening and diagnosis of ASD (Rellini et al., 2004). ABC has been widely applied in clinical institutions and scientific research and is currently one of the most mature assessment tools for ASD (Li et al., 2018).

### 2.3 Imaging acquisition

MRI scanning was attained at Shanghai Mental Health Center with a 3.0‐tesla Siemens Verio scanner. T1‐weighted anatomical MRI was obtained using 3D‐MPRAGE sequence: repetition time (TR) = 2530 ms; echo time (TE) = 2980 ms; inversion time = 1100 ms; flip angle (FA) = 7°; field of view (FOV) = 256 mm × 256 mm; matrix = 256 × 256; slice thickness = 1 mm, no gap; 192 sagittal slices; and acquisition time = 363 s. Resting‐state functional MRI (rs‐fMRI) data were acquired using an echo planar imaging sequence with the following protocols: TR/TE = 2000/30 ms, FOV = 220 mm × 220 mm, matrix = 64 × 64, FA = 90°, slice thickness = 3.2 mm, no gap, 43 interleaved transverse slices, 240 volumes, and acquisition time = 346 s. During the data acquisition, all participants were instructed to remain motionless, awake, and close their eyes (Biswal et al., 1995; Greicius et al., 2003; Zang et al., 2004).

### 2.4 Data preprocessing and processing

Data were processed and analyzed using Data Processing & Analysis for Brain Imaging (DPABI) (Yan et al., 2016) based on Matlab 2012a platform. The first 10 volumes were discarded because of the instability of the initial MRI signal and participants’ adaption to the noisy scanning environment. The remaining volumes were corrected for the acquisition delay between slices and then corrected for head motion using the Friston 24‐parameter model (Friston et al., 1996). Participants with head motion greater than 3 mm or over 3°of rotation in each axis were discarded. In order to further reduce the effect of motion on rs‐fMRI measures, we used the motion correction strategy suggested by Yan et al. (2016): identification of “bad” time‐points using a threshold of framewise displacement (FD) (Jenkinson ) > 0.2 mm, as well as one back and two forward neighbors, followed by modeling each “bad” time point as a separate regressor in the regression models (Jenkinson et al., 2002; Lemieux et al., 2007; Satterthwaite et al., 2013). We compared the head motions of the two groups using mean FD Jenkinson. The results showed that there was no significant difference between two groups in head motion (*p* = .055). Several nuisance covariates were regressed out, including the mean signal of WM/CSF and the Friston‐24 motion parameters. The data sets were then detrended and band‐pass filtered (0.01−0.08 Hz) to reduce the effects of low‐frequency drift and physiological high‐frequency respiration, and cardiac noise. ReHo value was acquired by counting the Kendall's coefficient of the time series of a given voxel with those of its neighboring voxels in original space (Zang et al., 2004). In order to lessen the whole‐brain effect, the ReHo value was divided by the mean value of their whole‐brain ReHo. For spatial normalization, the images were spatially normalized according to each subject's structural image using the DARTEL algorithm (Ashburner, 2007), and then resampled to 3 mm × 3 mm × 3 mm resolution. Subsequently, smoothing was carried out with a Gaussian filter of 4 mm full width at half maximum (FWHM) to reduce spatial noise.

### 2.5 Statistical analysis

The demographic and psychopathological characteristics were analyzed with SPSS software 18.0 for Windows. Two‐sample *t*‐test was used for testing the differences in age and FSIQ between adolescents with HFASD and TD controls. Pearson's chi‐square test was performed for comparing gender differences. The height threshold of statistical significance was set at a *p* value of .05.

Voxel‐wise two sample *t*‐tests were performed to explore differences in ReHo between adolescents with HFASD and TD controls, applying nonparametric permutation testing with 5000 permutations, with age, gender, FSIQ, and mean FD as covariates to reduce their potential influence on our results. The statistical significance was set at *p* < .001, correcting for family‐wise error (FWE) correction using threshold‐free cluster enhancement (TFCE). The brain areas with significant ReHo changes in individuals with ASD were extracted as inclusive masks in the subsequent correlation analysis.

For ReHo showing ASD‐related alterations, voxel‐wise correlation analysis was conducted to investigate its correlation with the psychopathological measures of the adolescents with HFASD, including the total scores and each component of ABC (Sensory, Relating, Body concept, Language, and Social self‐help), respectively. Participants’ age, gender, FSIQ, and mean FD were included as covariates to adjust for their potential confounding effects on relation between ReHo value and psychopathological variables. Only those ReHo values with significant group differences were examined, owing to an exploratory evaluation of these nonhypothesized regions. Correlations were assessed for significance using the nonparametric permutation testing with 5000 permutations and FWE‐TFCE multiple comparison‐corrected. A strict statistical significance of *p* < .001 with FWE‐TFCE correction was considered for the correlations between ReHo values and psychopathological data.

## 3 RESULTS

### 3.1 Participants’ demographic characteristics

Table 1 showed the demographic and clinical characteristics of ASD patients and TD controls in the current study. No significant differences appeared in age (*p* = .74), gender (*p* = .123), and FSIQ (*p* = .211) between two groups.

### 3.2 Disrupted ReHo in ASD

**FIGURE 1:**
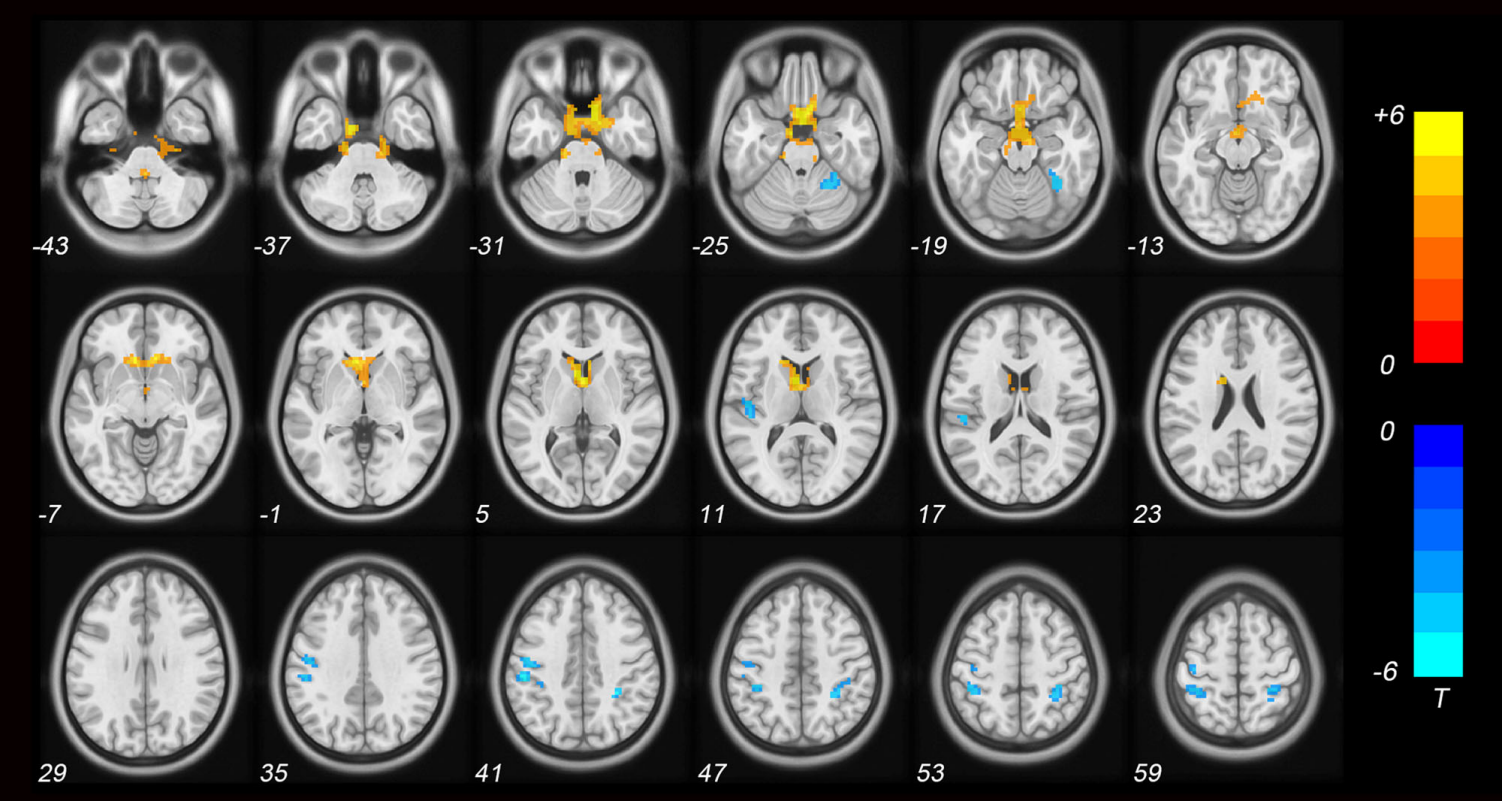
Brain regions with significantly altered ReHo in individuals with ASD compared to TD controls. Statistically significant differences in ReHo were defined as *p* < .001, corrected by TFCE and FWE after adjusting for age, gender, FSIQ, and mean FD. Warm color indicates that ReHo is higher in the ASD group than in the TD control group, and vice versa

Individuals with ASD exhibited increased ReHo in the bilateral anterior cingulate cortex (ACC), left caudate, right posterior cerebellum (cerebellar tonsil), and bilateral brainstem compared to TD controls (*p* < .001 for all, FWE‐TFCE‐corrected; Figure 1 and Table 2). Moreover, the ReHo values decreased in the left precentral gyrus, left inferior parietal lobule (IPL), bilateral postcentral gyrus, and right anterior cerebellum (culmen) (*p* < .001 for all, FWE‐TFCE‐corrected; Figure 1 and Table 3). Individual age, gender, FSIQ, and mean FD were used as covariates during the group comparisons. Subsequently, the correlation analysis in individual with ASD was carried out within the regions of ReHo alteration.

**TABLE 2:** Brain regions with increased ReHo in individuals with ASD compared with TD controls

		Cluster	MNI coordinates (mm)			
Region	Hemisphere	(voxel)	*x*	*y*	*z*	*t* Value
Limbic lobe						
ACC	Bilateral	60	8	21	–5	4.84
Sublobar						
Caudate	Left	104	–7	14	3	4.31
Cerebellum						
Cerebellar tonsil	Right	336	18	–39	–51	4.81
Brainstem	Bilateral	160	14	–24	–34	4.06

*Note*: Statistically significant differences in ReHo were defined as *p* < .001, corrected by TFCE and FWE after adjusting for age, gender, FSIQ, and mean FD.

Abbreviations: ACC, anterior cingulate cortex; MNI, Montreal Neurological Institute.

**TABLE 3:** Brain regions with decreased ReHo in individuals with ASD compared with TD controls

		Cluster	MNI coordinates (mm)			
Region	Hemisphere	(voxel)	*x*	*y*	*z*	*t* Value
Frontal lobe						
Precentral gyrus	Left	53	–41	–20	61	–4.36
Parietal lobe						
IPL	Left	41	–40	–39	50	–4.70
Postcentral gyrus	Left	43	–48	–15	15	–4.68
Postcentral gyrus	Left	61	–40	–38	58	–4.52
Postcentral gyrus	Right	60	34	–39	56	–4.56
Cerebellum						
Culmen	Right	69	33	–51	–21	–4.78

*Note*: Statistically significant differences in ReHo were defined as *p* < .001, corrected by TFCE and FWE after adjusting for age, gender, FSIQ, and mean FD.

Abbreviations: IPL, inferior parietal lobule; MNI, Montreal Neurological Institute.

### 3.3 Relationship between ReHo values and psychopathological characteristics

**FIGURE 2:**
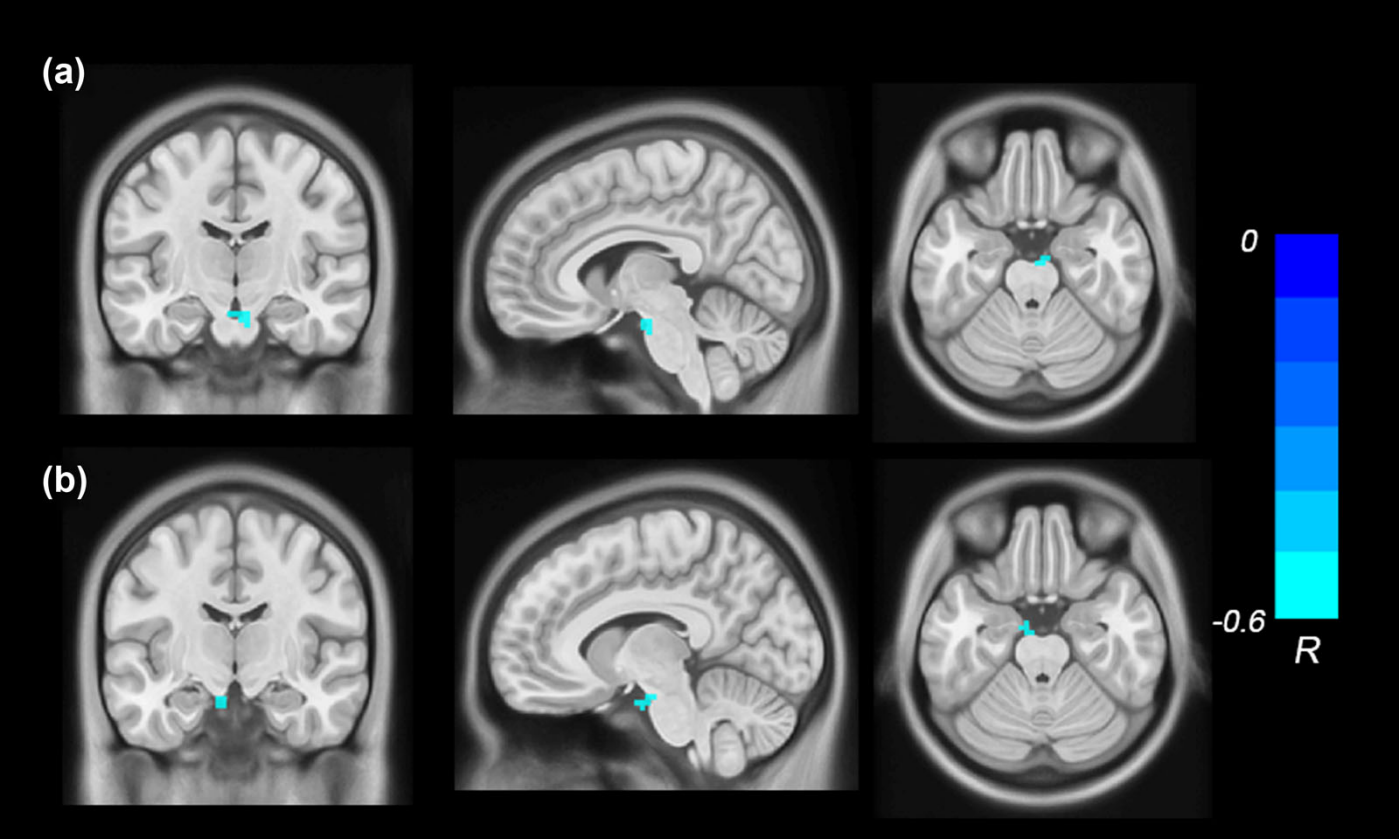
The correlation analysis showed the ABC total scores were negatively correlated with the ReHo values in the right brainstem (a), and the scores of Relating factor were negatively correlated with the ReHo values in the left brainstem (b). Significant correlations between ABC and ReHo were defined as *p* < .001, corrected by TFCE and FWE after adjusting for age, gender, FSIQ and mean FD. Cool color indicates negative correlation

In the ASD group, the ABC total scores were negatively correlated with the ReHo values in the right brainstem (*r* = −.54, *p* < .001, FWE‐TFCE‐corrected; Figure 2a), and the scores of Relating factor were negatively correlated with the ReHo values in the left brainstem (*r* = −.58, *p* < .001, FWE‐TFCE‐corrected; Figure 2b). The results of above voxel‐wise correlation analysis of the individuals with ASD are shown in Table 4.

**TABLE 4:** Significant correlations between ABC and ReHo in ASD

	Correlation			MNI coordinate			
	coefficient *r*	*p*	Anatomical region	*X*	*Y*	*Z*	Cluster size (voxel)
Total	–.54	<.001	Right brainstem	6	–15	–24	11
Relating	–.58	<.001	Left brainstem	–9	–12	–24	10

*Note*: Significant correlations between ABC and ReHo were defined as *p* < .001, corrected by TFCE and FWE after adjusting for age, gender, FSIQ, and mean FD.

Abbreviation: MNI, Montreal Neurological Institute.

## 4 DISCUSSION

In this study, we used the ReHo metric to examine local brain functional abnormalities in adolescents with HFASD and further investigated its correlation with symptom severity. Our results demonstrated that individuals with ASD exhibited increased ReHo in the bilateral ACC, left caudate, right posterior cerebellum (cerebellar tonsil), and bilateral brainstem and decreased ReHo in the left precentral gyrus, left IPL, bilateral postcentral gyrus, and right anterior cerebellum (culmen) compared to TD controls. Moreover, the ReHo value in the brainstem was negatively associated with the ABC total scores and the scores of Relating factor, respectively. The results documented that the ReHo abnormalities were present in ASD patients and might be involved in the clinical symptoms of this disorder.

Individuals with ASD showed widespread ReHo alterations in several brain areas, which was partially concordant with previous studies evaluating ReHo values in ASD patients compared to TD controls (Dajani & Uddin, 2016; Maximo et al., 2013; Paakki et al., 2010), suggesting that widespread disruption of local functional homogeneity might contribute to ASD pathophysiology. However, the convergence between these studies and the current study appeared to be modest, with only some roughly consistent results. These included local underconnectivity in ASD patients in postcentral gyrus and middle temporal gyrus, and overconnectivity in middle occipital gyrus (also detected by Paakki et al., 2010), and local underconnectivity in ASD patients in postcentral gyrus (also detected by Shukla et al., 2010). Besides, some other studies only found increased ReHo values in ASD (Jao Keehn et al., 2019; Li et al., 2018), or difference in ReHo values was detected predominantly in the right hemisphere (Li et al., 2018; Shukla et al., 2010). Several factors have been proposed to account for the inconsistent results, including the small sample size, different sample characteristics (age range, gender, or IQ), severity of symptoms, limited brain regions investigated, and differences in ReHo analysis, including special normalize, nuisance covariates regression, field strengths, and so on.

The precentral gyrus, situated in the posterior portion of the frontal lobe, is a very vital region involved in executing voluntary motor movements, also known as the primary motor cortex. The structural and functional abnormalities of the precentral gyrus, indicated by many neuroimaging studies (Abbott et al., 2018; Säisänen et al., 2019), have been considered to underlie the impairments of the afore‐mentioned function in ASD. Duffield et al. (2013) revealed that the finger‐tapping test was negatively correlated with precentral gyrus volume in ASD. Although the precentral gyrus is considered to be part of motor‐related cortex, activity in this area has previously been reported to associate with social–emotional functioning. Increased activitiy in precentral gyrus has been found when receiving empathic responses from others (Seehausen et al., 2016) and activity in this region is also related to self‐reported affective empathy in social versus nonsocial emotional scenes (Hooker et al., 2010). In the current study, reduced ReHo in the precentral gyrus was found in the ASD compared with TD group. The findings in the current study as well as previous imaging studies might point to an important role of the precentral gyrus in ASD.

In our study, the decreased ReHo were reported to be found in the IPL and postcentral gyrus, which are located in the parietal lobe. The IPL, which is located in the posterior region of parietal lobe, has been well‐known for its implication in visuospatial processing. Interestingly, several studies on visuospatial functioning have reported cognitive challenges for individuals with ASD (Mammarella et al., 2014; McGrath et al., 2012). For example, DeRamus et al. (2014) used a simple visual task of location detection to examine the visuospatial processing and fMRI to explore the location of brain dysfunction while detecting the location of objects, and revealed that ASD patients displayed a relatively intact visuospatial processing and significantly greater activation in the IPL while detecting the location of objects when compared with TD controls. As mentioned earlier, the bilateral postcentral gyrus showed decreased ReHo in individuals with ASD. The postcentral gyrus is the site of the primary somatosensory cortex, which is concerned with the main sensory reception of the sense of touch. Lee Masson et al. (2020) indicated that ASD patients may experience changes in touch, which is related to the sense of contact between people. Sensory problems are identified as core symptoms of ASD (American Psychiatric Association, 2013), which make a contribution to social and repetitive behavior deficits (Foss‐Feig et al., 2012). Consistent with findings of our study, Mizuno et al. (2019) suggested that developmental delay in the postcentral gyrus increased the risk for sensory processing deficits. In current study, the IPL and postcentral gyrus showed decreased ReHo in ASD, which may contribute to the neurobiological basis of abnormal sensory perception in ASD.

Additionally, we also found that compared with TD controls, ASD patients had significantly increased ReHo in the bilateral ACC compared with TD controls. The ACC, purportedly, has been involved in various social–emotional and cognitive functions (Allman et al., 2001). Other studies also showed abnormalities in the ACC in ASD. Several task‐based fMRI studies have showed activation abnormalities of ACC along with poorer task performance in individuals with ASD (Di Martino et al., 2009). Greimel et al. (2013) reported reduced GMV in the ACC in ASD patients. Interestingly, a magnetic resonance spectroscopy (MRS) study has also reported decreased brain metabolites in the ACC in ASD patients (Auvichayapat et al., 2020). Therefore, the ACC is likely to be a promising target region for the neural mechanisms study of ASD.

In our study, increased ReHo was also reported in the caudate, which is an important area involved in motivation and evaluation of action outcomes (Delgado et al., 2004; Grahn et al., 2008). This is not surprising because the decrease in interest and motivation for social interaction at the behavioral level (Chevallier et al., 2012; Morrison et al., 2017) and the decrease in caudate nucleus activity for social and nonsocial reward tasks at the neurological level have been both observed in ASD (Clements et al., 2018; Delmonte et al., 2012). Several volumetric MRI studies have reported GMV alterations in the caudate and their association with restricted and repetitive behaviors in children and adults with ASD (Hollander et al., 2005; Qiu et al., 2016). Thus, it can be seen that caudate is abnormal in both structural and functional metrics and that it may be the neural bases for the causes and symptomatology of ASD.

The cerebellum is one of the most consistent sites of aberrance in ASD, which have been associated with ASD for more than two decades (Fatemi et al., 2012; Wolff et al., 2017). The notion that cerebellum is only concerned with fine motor function has been outdated; there are a lot of evidences that the cerebellum is related to higher cognitive functions, including language, emotional regulation, episodic, and working memory (Hampson & Blatt, 2015; Qin et al., 2019). The cerebellar tonsil is situated in the posterior lobe, which plays a vital role in cognition and emotional regulation (Stoodley & Schmahmann, 2009). The culmen, situated in the portion of the anterior vermis, is considered correlated with frontal cognitive functions in imaging studies (Li et al., 2011). In our study, increased ReHo was also observed in the right cerebellar tonsil of individuals with ASD, which was documented to be linked to the executive control of working memory by preventing irrelevant information from getting into working memory (Baier et al., 2014). In addition, we also found reduced ReHo in the right culmen, which was reported coactivated with frontal cognitive areas in sequencing learning, memory retrieval, and verbal working memory (Desmond & Fiez, 1998). Therefore, our findings may give a preliminary evidence to support the engagement of cerebellum in regulating cognition in ASD.

In this study, increased ReHo in the bilateral brainstem may be linked to autistic symptoms in individuals with ASD, particularly in the relating domain. Specifically, a range of autism symptoms, such as social interaction impairments combined with restrictive and repetitive behaviors and interests, may be related to sensory disturbance (Sinclair et al., 2017). ASD individuals also exhibit disruptions of sensory processing, which are manifested in hypersensitivity, avoidance of sensory stimulation, and/or sensory search behavior (Sinclair et al., 2017). Furthermore, sensory processing difficulties are considered to stem from the brainstem (Dadalko & Travers, 2018; Seif et al., 2021). The brainstem is a structure juncture that is linked with the cerebrum, cerebellum, and spinal cord. Abnormal brainstem development and/or brainstem processing delay have a cascading effect on the higher centers of the brain (Seif et al., 2021). Indeed, the brainstem volume reduction has been consistently reported in studies on brainstem volume in ASD (Fredo et al., 2014; Herbert et al., 2003). Several studies have also observed the white matter reduction of brainstem in ASD (Hanaie et al., 2016; Toal et al., 2010). Additionally, a study investigating structural and functional connectivity of the mesolimbic reward pathway showed structural abnormalities in the white matter tracts that reciprocally connect the nucleus accumbens of the striatum and the ventral tegmental area of the midbrain (Supekar et al., 2018). Furthermore, they reported that structural abnormalities are accompanied by abnormal functional interactions between nucleus accumbens and ventral tegmental area in response to social stimuli (Supekar et al., 2018). The mesolimbic reward pathway is a central circuit for the brain to process reward value (O'Connell & Hofmann, 2011). A well‐known ASD theory assumes that ASD individuals find social stimuli less rewarding than their TD peers, resulting in impaired social interaction (Chevallier et al., 2012). In our study, we also found ReHo alterations in the brainstem and association with the ABC total scores and the scores of Relating factor, respectively. Taken together, there is increasing evidence regarding the important role of brainstem disruptions in ASD. Studying how autistic symptoms stem from the aberrance of brainstem may help understand the neurobiological causes of ASD and may ultimately identify biomarkers for early diagnosis and intervention to improve the outcome of the affected individuals.

Several limitations should be considered in our study. First, the sample size in our study was still relatively small on account of recruitment difficulties of adolescents with HFASD, which may have reduced statistical power. The current study has a strictly limited inclusion criteria (i.e., adolescents with HFASD) to increase statistical power and reduce variability. However, since ASD is a heterogeneous disease with multiple risk factors and causes, our sample may not be sufficiently homogeneous. For that reason, future studies with larger samples of individuals falling on different subgroups of the ASD spectrum are desirable to obtain a better description of ReHo changes that are involved in the symptomatology of ASD. In addition, due to the cross‐sectional design we cannot elucidate the developmental‐related characteristics of these brain alterations in individuals with ASD. Specifically, several researches have reported that age may influence the cerebral ReHo in the resting state (Long et al., 2017; Sheng et al., 2016). Age difference may cause the inconsistent results in examining the ReHo of ASD individuals, so future investigations will be needed to examine how the ReHo longitudinally change with age in ASD compared to TD controls. Furthermore, our study used strict inclusion criteria and control for different variables, such as age and IQ, and hence, the generalizability of our findings is inevitably reduced. Last but not the least, the individuals who were not able to finish all the tests and keep the head still in an MRI scanner were excluded from the study and, therefore, raise the possibility that our findings are not representative of all individuals with ASD.

In summary, with fewer confounders, our findings indicate that ASD patients have altered ReHo values in numerous regions, suggesting the involvement of ReHo abnormalities of ASD. Moreover, the ReHo abnormalities of the brainstem were related to the symptom severity, suggesting that ReHo abnormalities may be associated with the psychopathology of ASD. The findings from this study seem to provide an empirical and theoretical basis for exploration for biological markers and further targeted therapy of ASD. While we speculated that the ReHo aberrance of brain regions may be the neuropathological mechanism underlying the symptomatology of ASD, the mechanisms still remain unknown. Thus, the picture that emerges is that ReHo alterations require replication in larger samples in individuals with ASD.

## CONFLICT OF INTEREST

The authors declare that they have no conflict of interest.

## FUNDING

This work is partly supported by the Scientific Research Project of Shanghai Municipal Health Commission (20194Y0071), the Shanghai public health system construction 3‐year action plan (GWV‐10.1‐XK19), and the National Key Development Program (2017YFC1309903).

## AUTHOR CONTRIBUTIONS

Xiaoxin Zhao, Wenqing Jiang, Yan Li, and Yasong Du had full access to all study data and developed the study concept and design. Shuyi Zhu, Peipei Cheng, Yang Cao, Yuxiong Lin, and Zhixin Sun collected the data and aided in data interpretation. Xiaoxin Zhao took responsibility for accuracy of data analysis and drafted the manuscript. Yasong Du undertook the critical revision of the manuscript. The authors read and approved the final manuscript.

### PEER REVIEW

The peer review history for this article is available at: https://publons.com/publon/10.1002/brb3.2693.

## Data Availability

The data that support the findings of this study are available from the corresponding author upon reasonable request.
